# Critical Nodes in River Networks

**DOI:** 10.1038/s41598-019-47292-4

**Published:** 2019-08-01

**Authors:** Shiblu Sarker, Alexander Veremyev, Vladimir Boginski, Arvind Singh

**Affiliations:** 1 Department of Civil, Environmental and Construction Engineering, University of Central Florida, Orlando, Florida 32816 USA; 2 Department of Industrial Engineering and Management Systems, University of Central Florida, Orlando, Florida 32816 USA

**Keywords:** Engineering, Hydrology

## Abstract

River drainage networks are important landscape features that have been studied for several decades from a range of geomorphological and hydrological perspectives. However, identifying the most vital (critical) nodes on river networks and analyzing their relationships with geomorphic and climatic properties have not yet been extensively addressed in the literature. In this study, we use an algorithm that determines the set of critical nodes whose removal results in maximum network fragmentation and apply it to various topologies of simulated and natural river networks. Specifically, we consider simulated river networks obtained from optimal channel network (OCN) approach as well as extracted networks from several natural basins across the United States. Our results indicate a power-law relationship between the number of connected node pairs in the remaining network and the number of removed critical nodes. We also investigate the characteristics of sub-basins resulted from the removal of critical nodes and compare them with those of central nodes (in the context of betweenness centrality) for both natural basins and OCNs with varying energy exponent γ to understand vulnerability and resilience of river networks under potential external disruptions.

## Introduction

River networks depend on external forcings such as climate and tectonics^1–12^. These dendritic networks serve as primary pathways for transport of sediment, water, and other environmental fluxes and provide necessary ecosystems to a variety of ecologic and biotic activities^13–19^. However, these networks are facing significant threats under changing climate and anthropogenic activities. Therefore, the knowledge of the topologic structure and dynamics of river networks is crucial for better understanding of their emergence and evolution under change as well for prediction and management of environmental fluxes operating upon them^1,13–16,20–26^.

Branching patterns of river networks have been argued to exhibit complex topology and share similar properties such as scaling and self-similarity with other complex systems^27–30^. Recent theoretical and empirical evidences suggest that branching complexity is a key to maintaining meta-population stability^19^ and in driving biodiversity patterns^18^. A common way to quantify network complexity is via graph-theoretical approaches that have been used extensively in characterizing networks from diverse fields including but not limited to social networks, transportation networks, communication networks, and networks from computer science and mathematics^29,31–35^.

Although there are many different approaches for quantifying node importance (see, e.g., Borgatti^36^), in this study we focus on the one which is based on the effect of node removal to the network structure. The corresponding problem is known as the *critical node detection* problem (see Lalou *et al*.^37^ for a more detailed discussion on this topic). Specifically, we consider the number of connected pairs of nodes in the remaining network as a measure of “integrity” or “connectivity” of river networks and find the set of nodes whose removal minimizes this measure. Despite significant research on river network characterization using graph theory^7,38–40^, to the best of our knowledge, there are no previous studies that focus on critical node identification on river network topologies.

As a solution approach for finding exact locations of critical nodes, we use the linear integer programming problem formulation developed in Veremyev *et al*.^41^ due to its simplicity of implementation and effectiveness on the considered networks. In addition, since all river networks investigated here are trees, we demonstrate that the considered critical node identification problem is equivalent to the problem of finding a group of nodes with the highest *group betweenness centrality*^42^, which can be interpreted as a quantitative measure of the role of a group of nodes as intermediaries in the process monitoring and control of flow in a network^43–45^. Note that, as discussed in more detail below, there is a difference between the related notions of the highest *group betweenness centrality* and a group of nodes where *each* node has a high “individual” betweenness centrality. Therefore, we distinguish the concepts of “critical” and “central” nodes throughout the paper. We investigate the locations of the most influential nodes obtained from both critical nodes and central nodes approaches and their role on the generation of sub-basins via fragmentation of river network topology for various simulated and real river networks. The implications of these results are discussed from a network protection perspective under potential natural or man-made disruptions.

## Results and Discussion

### Simulated and natural river networks

In this study, we generate river networks using optimal channel network (OCN) approach. The OCNs recreate several topologic and geometric properties commonly observed in real river networks. They have been explored extensively in the past and recent studies from a range of hydrologic and geomorphic perspectives^7,21,46–49^. In general, OCN modeling is based on the local minimization of the topologic energy defined as $E={\sum }_{i=1}^{N-1}\,{L}_{i}{q}_{i}^{\gamma }$, where ${L}_{i}{q}_{i}^{\gamma }$ represents the energy dissipated in the *i*^*th*^ link of the network, and *L*_*i*_ and *Q*_*i*_ are its length and discharge, respectively^30^. The energy exponent *γ*, varies between 0 and 1, characterizes the mechanics of erosional processes and defines branching pattern of the channel network^30^. For simplification, the topologic energy was computed assuming the unit distance between two adjacent nodes and a uniform precipitation over the entire simulated basin was applied^7,38^. Here, we generate channel networks for different values of *γ* (from 0.1 to 0.9; representing varying geomorphic processes) keeping the initial random tree network the same for each simulation.

**Figure 1:**
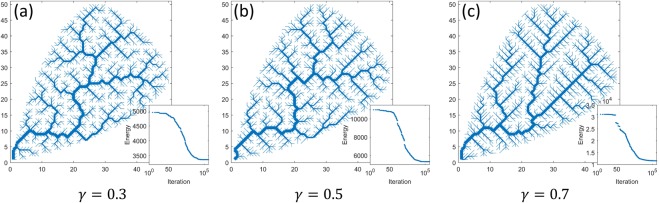
Synthetic river networks generated using OCN model within a defined basin boundary and uniform rainfall for (**a**) $\gamma =0.3$, (**b**) $\gamma =0.5$, and (**c**) $\gamma =0.7$. The size of the square grid used was 50 × 50. The line thickness represents the channel order. The bottom right subplots in each panel show the total energy expenditure as a function of number of iteration.

The OCN basins were simulated on an arbitrary shape mimicking real basins using an initial square grid of size $N\times N$ nodes, where $N=50$ (see, Abed-Elmdoust *et al*.^7^). Figure 1 shows simulated OCNs within a defined basin boundary obtained under uniform rainfall for $\gamma =0.3$ (Fig. 1a), $\gamma =0.5$ (Fig. 1b), and $\gamma =0.7$ (Fig. 1c). Note that the networks shown here corresponded to the iteration with minimum energy $(\,\sim \,2\times {10}^{5})$.

We also extracted river networks from six different natural basins located in different states across the United States (see Table 1 for details). The river networks were computed from 10 m resolution digital elevation models (DEMs) using same area threshold. The DEMs corresponded to varying hydrologic and geomorphic conditions^9,50^ (Table 1) and were downloaded from National Elevation dataset website (https://viewer.nationalmap.gov/basic/).

**Table 1:** Climatic and geomorphic properties of natural basins.

Basins	Drainage area (km^2^)	Total channel length (km)	Drainage density (1/km)	Maximum channel order	Average temperature (°F)	Average annual precipitation (rainfall-inch)	Total number of nodes
California	1931.05	387.78	0.201	4	64.1	12.83	76
Florida	4166.05	967.82	0.232	4	68.3	56.52	166
Texas	3365.24	761.14	0.226	4	64.3	40.97	128
Virginia	2215.18	585.17	0.264	4	56	43.11	92
South Dakota	4571.53	1350.91	0.295	5	43	21.76	188
Washington	1439.84	364.205	0.252	3	52.65	37.13	72

### Centrality and node importance

Centrality is one of the most fundamental concepts in network analysis which is used to quantify the “importance” or “influence” of a node to the network structure. Although various centrality measures have been proposed and investigated in many different contexts^51,52^ including network vulnerability analysis^53,54^, in this paper we focus on one particular centrality measure, betweenness centrality, due to its natural interpretation in the context of nodes “controlling” the environmental fluxes (e.g. water, sediments, nutrients) through river networks, as well as due to its direct relation to the critical node detection problem which we will state formally later. Specifically, we identify the locations of the most *central* nodes according to the betweenness centrality measure and compare them with the locations of the most *critical* nodes.

Betweenness centrality (BC) is a commonly used topological measure for identifying location and evaluating influence of a node on the overall network^55^. It is a local measure (assigns a score for each node) and is based on the number of shortest paths captured by each node traversing through it from the entire network. For a given network with nodes *N*, if ${n}_{st}(u)$ is the number of shortest paths from node *s* to node *t* that pass-through node *u* and *n*_*st*_ is the total number of shortest paths from node *s* to node *t* from the entire network, then BC (or influence) score (not scaled) of node *u* can be mathematically expressed as:

1 $$C(u)=\sum _{s,t\ne u}\,\frac{{n}_{st}(u)}{{n}_{st}}.$$

**Figure 2:**
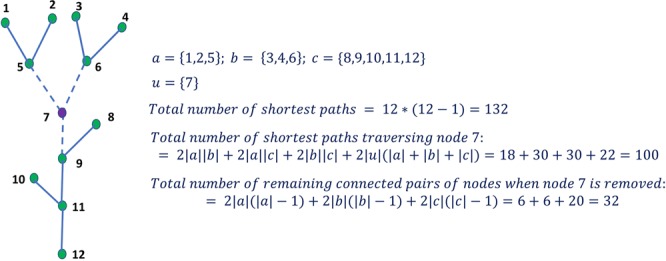
Schematic example of a tree network to illustrate the concepts of central and critical nodes. Here, size of the group is considered to be equal to 1 (|*S*| = 1) and *a*, *b*, and *c* are the remaining connected components (fragmentation) after the removal of node 7. Therefore, in this case, local centrality measure and the group measure are the same. However, in the case of group size |*S*| > 1, the group BC measure is simply fraction of the shortest paths captured by at least one of the nodes in that group.

Figure 2 shows a hypothetical network with 12 nodes and illustrates the concept of BC. For example, out of $12\ast (12-1)=132$ shortest paths among all pairs of nodes (note that there is only one shortest path between any pair of nodes in this network), the number of shortest paths captured by node *u*, here $u=7$, (i.e., shortest paths containing node *u*) is 100, hence, $C(u)=100$. Notice, in this example, BC score (scaled) of node 7 is =100/132; this is an individual or local BC measure for node 7. Most central nodes can be identified on any network based on the individual BC score.

**Figure 3:**
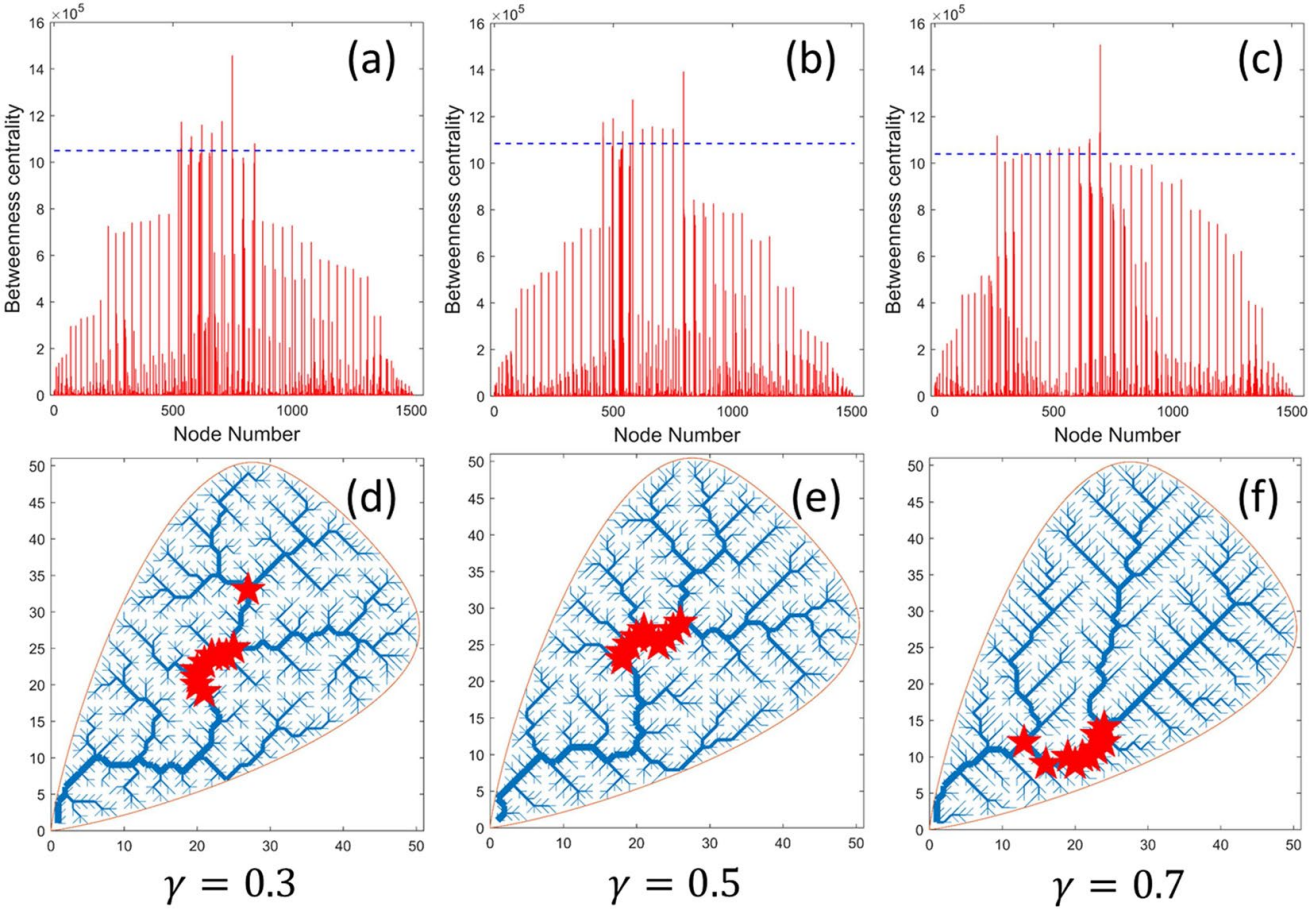
Betweenness Centrality (undirected) for (**a**) $\gamma =0.3$, (**b**) $\gamma =0.5$, and (**c**) $\gamma =0.7$. The dashed horizontal line in (**a**–**c**) shows the threshold level above which 10 most central nodes were selected. Plots (**d**–**f**) show locations of the 10 most central nodes superimposed on the river network for $\gamma =0.3$, 0.5, and 0.7, respectively.

To identify the locations of the most central nodes and their influence (strength) on the network, we computed BC for each node in the entire network for varying energy exponent *γ* (note that the number of shortest paths between any pair of nodes *s*, *t* is ${n}_{st}=1$ if a graph is a tree which is the case in our setup). Figure 3 shows, as an example, the computed node-wise BC (in particular, undirected BC) using equation (1) for $\gamma =0.3$ (Fig. 3a), $\gamma =0.5$ (Fig. 3b), and $\gamma =0.7$ (Fig. 3c). Assuming that we are interested in identifying 10 most central nodes on the network, we selected 10 nodes for each *γ* above a threshold value represented by dashed lines (Fig. 3, top panels). Figure 3(d–f) show locations of the 10 most central nodes superimposed on the OCN for $\gamma =0.3$, 0.5, and 0.7, respectively. Also note that although we have computed networks and their characteristics for $\gamma =0.1-0.9$, we only show results for $\gamma =0.3$, 0.5, and 0.7 due to space considerations.

### Critical node identification

Critical node identification (CNI) problem deals with the optimal deletion of nodes from a network to maximize the network fragmentation. This can be achieved by minimizing the size of the largest remaining connected components or pair-wise connectivity, i.e., the total number of node pairs connected by a path^41,56,57^. The pair-wise connectivity is a network disruption metric which can be used for understanding network vulnerability (i.e., optimal response of a network to an external attack) and protection (i.e., network defense) purposes. Therefore, critical nodes represent a group (subset) of nodes that are crucial for maintaining the integrity of a network^37^.

In this study we use the graph-theoretic framework and integer programming formulations to identify critical nodes on the river network topology (see Methods for details). This framework can be used to detect critical nodes on a tree network. Since river networks exhibit tree like structures which can be delineated numerically using stream ordering schemes^58–62^, of interest would be to explore locations of critical nodes on these networks.

Moreover, since the considered river networks are trees, any pair of nodes is connected by exactly one shortest path. Hence, a pair of nodes becomes disconnected if a node belonging to the corresponding shortest path is removed. Thus, the number of pairs of nodes that can be disconnected by removal of one node *u* is equal to the BC score *C*(*u*) of a corresponding node.

Therefore, the detection of critical nodes in our network topology by minimizing the pairwise connectivity is equivalent to identifying most influential (central) group of nodes with the highest *group betweenness centrality*^42^ with same predefined size as number of critical nodes. In particular, the pairwise connectivity is the inverse of network fragmentation and the set of critical nodes is equivalent to a group of nodes with maximum group betweenness centrality which is a group centrality measure as opposed to individual node measure (i.e. BC).

For a group of nodes $S\subseteq N$ in a given undirected graph *G*, the group betweenness centrality of *S* in *G* can be defined as^43,44^:

2 $$C(S)=\sum _{s,t\in N}\,\frac{{n}_{st}(S)}{{n}_{st}}.$$

This group betweenness centrality measure is based on the number of the shortest paths ${n}_{st}(S)$ between any pair of nodes $(s,t)$ in a graph *G* that pass through at least one of the node in a group *S*, thus relating pairwise connectivity with group betweenness centrality. Therefore, the critical nodes act together as a *group capturing the highest number of shortest paths* between all pairs of nodes in the network.

In other words, in the group betweenness centrality maximization formulation, the group *S* is the set of critical nodes whose removal fragments the network into a number of connected components that can be inferred as sub-basins of the river network *G*. In the example shown in Fig. 2, *a*, *b* and *c* are the remaining connected components due to the deletion of node 7. For a group size of 1, node 7 is the only node whose removal provides minimum pairwise connectivity (32 = 6 + 6 + 20 connected node pairs remain within connected components *a*, *b* and *c*, respectively) or maximum fragmentation of the network. For this group size, critical node is also the most central node with the highest BC score, as the number of shortest paths traversing through node 7 plus the number of remaining connected node pairs when node 7 is removed is 132, which is the total number of shortest paths among all pairs of nodes. Since, in the case of a tree network, any pair of nodes is connected by one and exactly one shortest path as there is no loop in the tree network, in such a case, critical nodes are equivalent to group of nodes with highest BC score.

**Figure 4:**
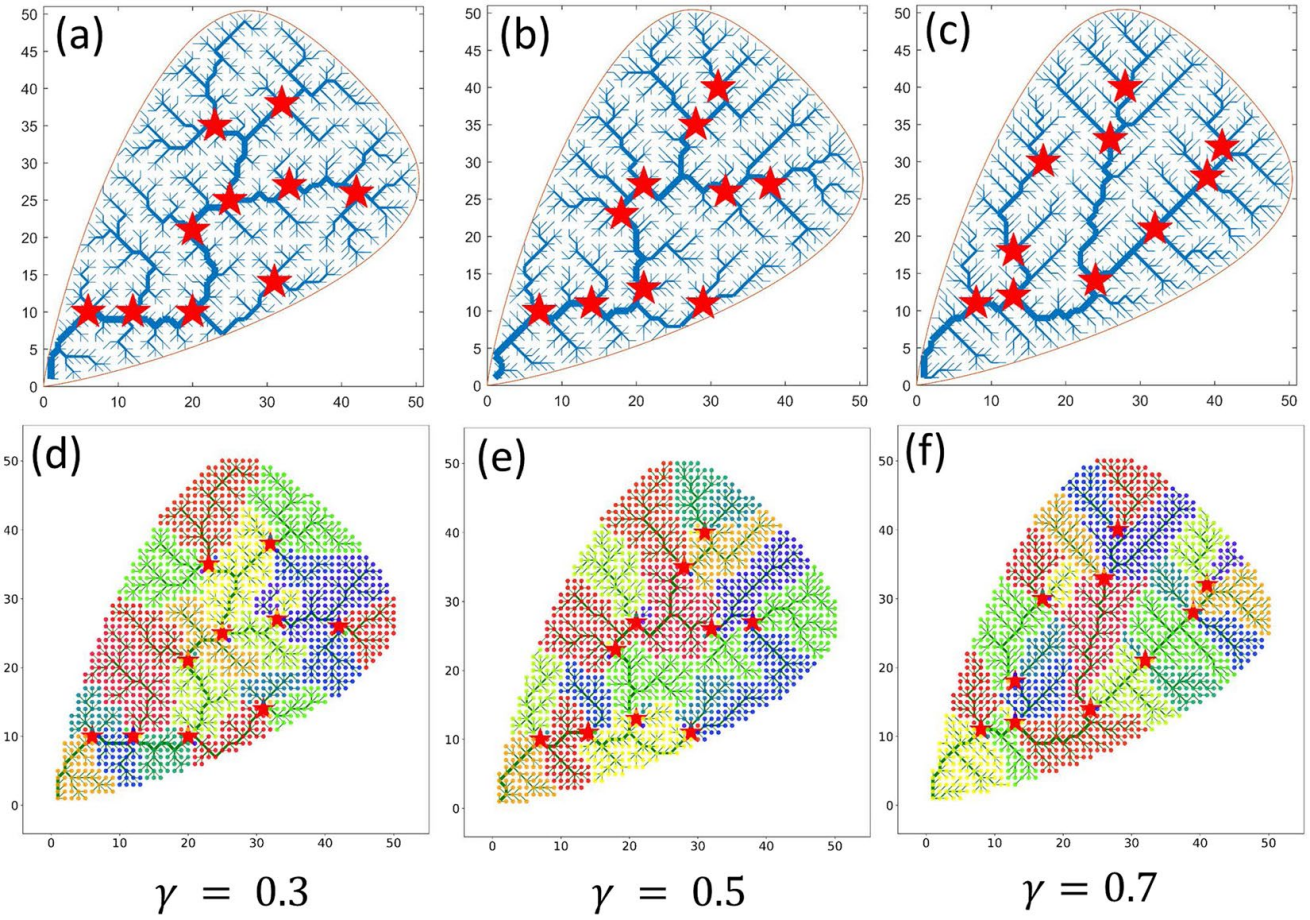
Locations of critical nodes on the synthetic networks for (**a**) $\gamma =0.3$, (**b**) $\gamma =0.5$, and (**c**) $\gamma =0.7$. The associated sub-basins emerged as a result of the removal of the critical nodes can be seen in figures (**d**–**f**) for $\gamma =0.3$, 0.5, and 0.7, respectively.

**Figure 5:**
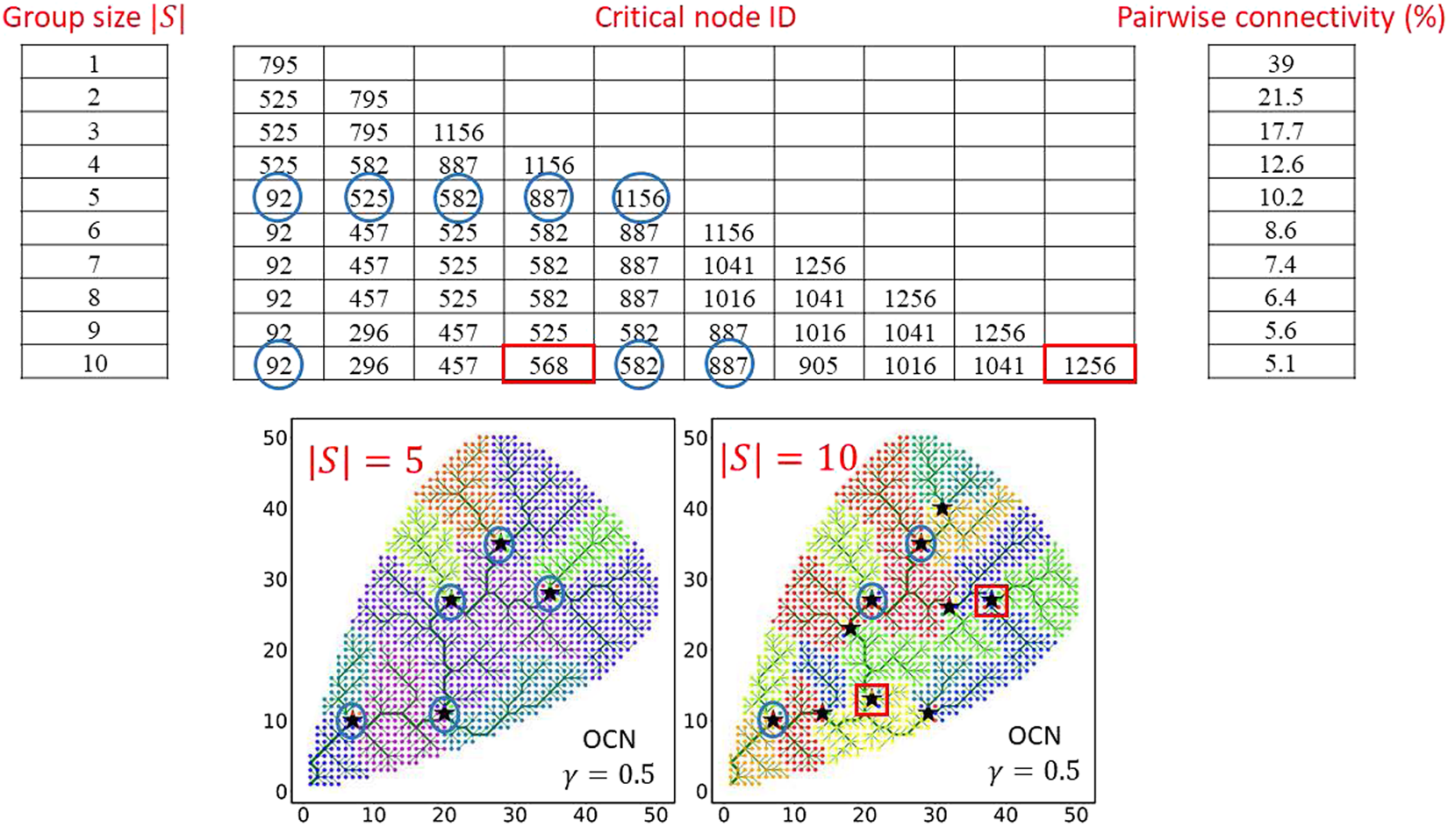
Comparison of critical node locations on a synthetic OCN corresponding to $\gamma =0.5$ for two different group sizes (i.e. |*S*| = 5 and |*S*| = 10). The critical nodes ID is also presented for visualization purposes. Among 5 critical nodes (shown as blue circles) in |*S*| = 5 (bottom left panel), 3 are common in |*S*| = 10 (bottom right panel), however, 2 critical nodes (shown as red boxes) change their locations in the group size represented by |*S*| = 10.

Figure 4(a–c) depicts the locations of critical nodes on the synthetic networks generated by OCN approach for $\gamma =0.3$, $\gamma =0.5$, and $\gamma =0.7$ (Fig. 4a–c, respectively) and the associated sub-basins emerged as a result of the critical nodes deletion (Fig. 4d–f). The definition of equation (2) also implies that, for $|S|=1$, group BC becomes individual node BC. In other words, if one looks for a *single* “most important” node, then the most central node in a river network is also the most critical node. However, the location of the critical node is dependent on the group size |*S*|. The example shown in Fig. 5 illustrates the locations of the critical nodes based on the group sizes of $|S|=5$ (circle) and $|S|=10$ (stars) on the same synthetic network. It can be seen that the critical nodes for $|S|=5$ are not necessarily a subset of critical nodes for $|S|=10$. There are 3 critical nodes out of total 5 that are common in the group size $|S|=5$ and $|S|=10$, indicating that the location of the critical nodes is dependent on the group size. In this study, we use a user defined group size to minimize pairwise connection of the network under node removal. In short, critical nodes for a given group size (e.g. $|S|=5$) is not necessarily a subset of critical nodes for larger group size (e.g. $|S|=10$). However, note that, one can constrain an optimization problem where a group of identified critical nodes can be fixed in case new critical nodes need to be identified on the network (e.g. for a larger group size).

**Figure 6:**
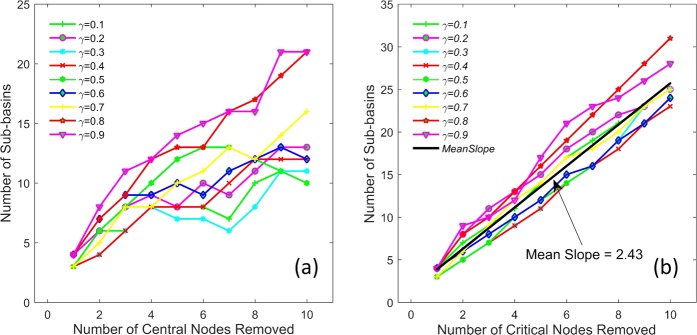
Fragmentation of network resulting in sub-basins due to removal of the (**a**) most central nodes and (**b**) the most critical nodes. Notice that the removal of critical nodes results in approximately linear slopes between the number of sub-basins and number of nodes removed as a function of *γ*.

To investigate the characteristics of sub-basins formed by the removal of critical nodes from the network as a function of *γ*, we analyze the number of fragments (sub-basins) from the modeled channel networks obtained using OCN approach for different *γ* values. These results are shown in Fig. 6 and discussed in the following sections.

### Central nodes vs critical nodes

Comparing locations of a set of most central nodes with most critical nodes on a network (Figs 3 and 4), it can be seen that the central nodes tend to be more clustered (localized) than critical nodes. To further understand and compare the characteristics of sub-basins induced by the central nodes and critical nodes, we analyze the number of sub-basins versus number of nodes removed. Figure 6 shows the fragmentation of the network resulting in sub-basins due to the removal of the most central nodes (Fig. 6a) and the most critical nodes (Fig. 6b). Here, 10 most central and most critical nodes are considered for comparison. Unlike in Fig. 6a, number of sub-basins follow a linear trend when plotted against the number of critical nodes removed for varying *γ* with an average slope ~2.43 ± 0.26. Moreover, the number of sub-basins (fragmentations) induced by critical node deletion is much larger in the case of critical nodes than central nodes. For example, excluding $\gamma =0.8$ and $\gamma =0.9$ (Fig. 6a), on average the number of sub-basins generated in the case of central nodes is roughly half of that as in the case of critical nodes, as a result of a closer node localization in the case of most central nodes.

**Figure 7:**
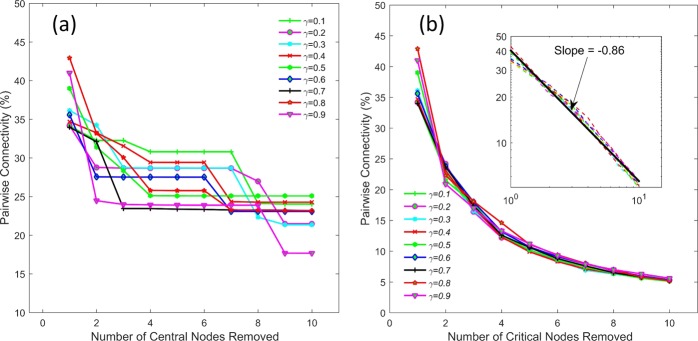
Pairwise connectivity of the network for different *γ* values due to removal of the most central nodes (**a**) and the most critical nodes (**b**). Inset in (**b**) shows the pairwise connectivity for critical nodes on a log-log plot, suggesting power-law behavior.

Figure 7 shows the pairwise connectivity (i.e. number of connected node pairs in the remaining network) of the network for different *γ* values due to removal of the most central nodes (Fig. 7a) and the most critical nodes (Fig. 7b). A few observations can be made from Fig. 7. i) The pairwise connectivity (PC) shows higher variability as a function of *γ* for central nodes, whereas it almost remains same for critical nodes. ii) Overall, PC is higher for central nodes for larger number of nodes removed compared to critical nodes. iii) While the PC decreases with increasing *γ* for both most central and critical nodes, a clear power-law decay is observed in the case of critical nodes with slope parameter approximately ~−0.86 (inset in Fig. 7b). Figures 6 and 7 also suggest that the removal of subsequent central nodes on the list of most central nodes captured a lot of the same paths that were already served by the removal of previous nodes on the list. On the other hand, the group measure of BC has the ability to resolve this issue.

Based on above observations and defining vulnerability as the maximum fragmentation of a network, we argue that critical nodes, as opposed to central nodes, provide a more consistent measure to quantify the vulnerability of a river network under node disruptions.

### Critical nodes on natural river networks

**Figure 8:**
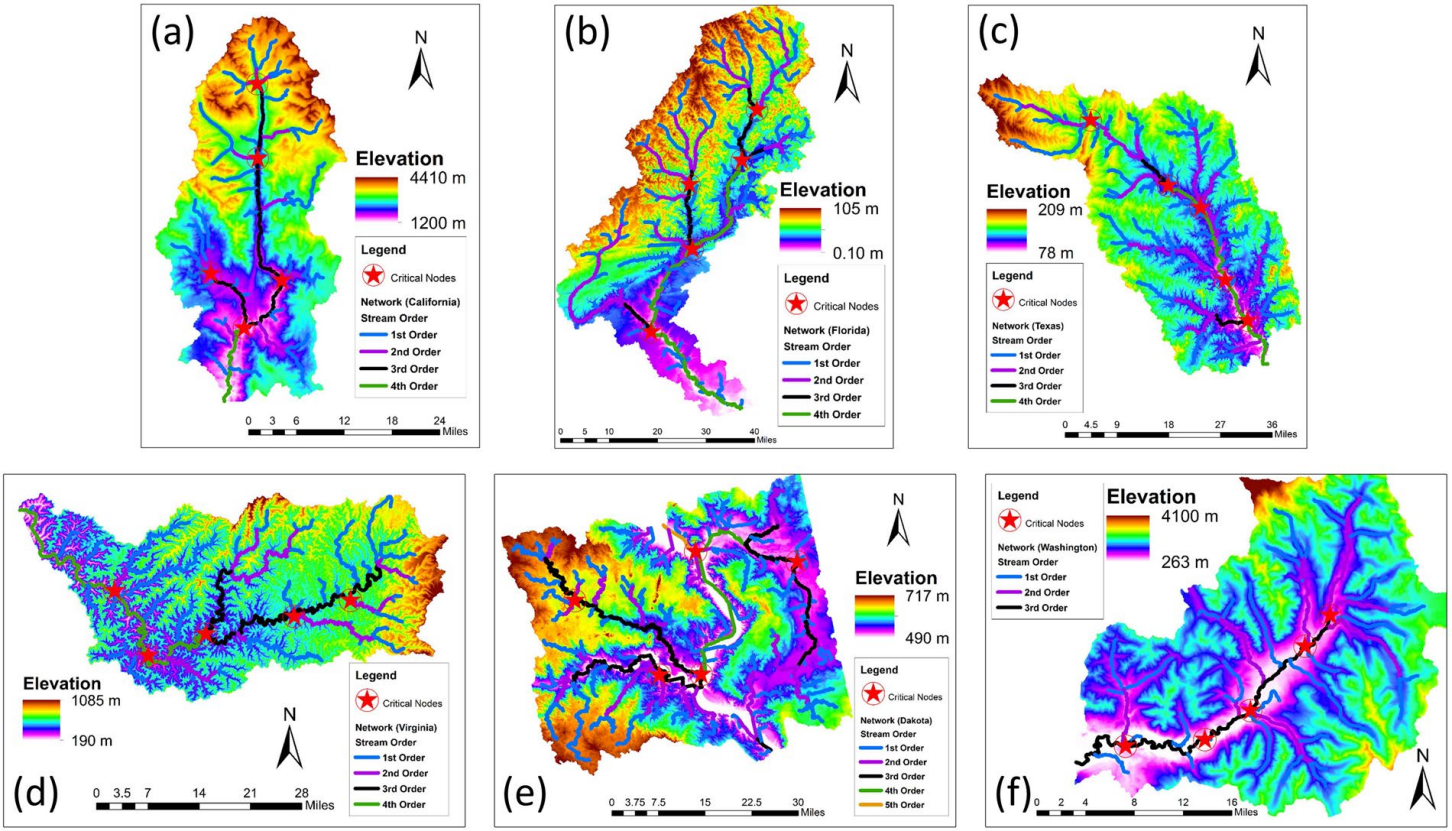
DEMs and superimposed river networks for six natural basins used in this study. 5 out of 10 identified critical nodes are also superimposed on the network for visualization purposes. The resolution of collected DEMs was 10 m. The channel order based on the Horton-Strahler^59^ ordering scheme is shown with different colors.

Natural river networks were extracted from 10 m resolution DEMs from six different regions across the United States (see Fig. 8 and Table 1). The networks were extracted based on same threshold flow accumulation value for channel initiation^50^. Similar to simulated networks, 10 critical nodes were identified using CNI algorithm discussed above and in Methods and were superimposed on the network for visualization purposes (Fig. 8). As can be seen from Fig. 8, the identified critical nodes are scattered around the network on the basin. Note that for visual perception purposes, only five critical nodes are shown on each network in the figure; however, both pairwise connectivity and number of sub basins were computed based on 10 most critical nodes.

**Figure 9:**
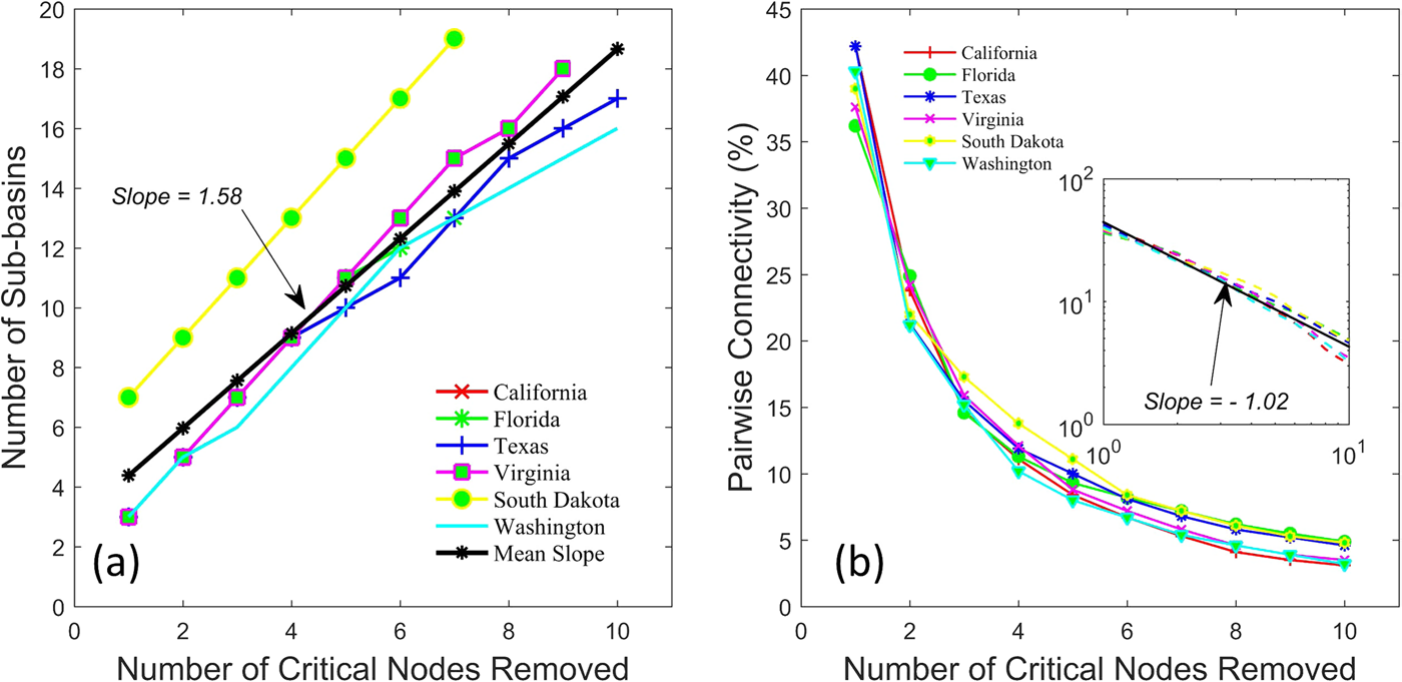
(**a**) Number of sub-basins emerged and (**b**) pairwise connectivity (%) as a function of number of critical nodes removed for different natural basins. The inset in (**b**) shows pairwise connectivity on a log-log scale. The numerical values represent the average slope fitted to linear regression lines in (**a**) and to the power-law relationships shown in inset in (**b**).

Figure 9a shows the number of sub-basins generated as a function of number of critical nodes removal for different natural basins, exhibiting approximately linear relationship with removed critical nodes. However, a significant variability is observed between basins, in particular for a larger set of critical nodes removed. Also, note a higher fragmentation in case of river network from South Dakota; this may be due to higher drainage density (ratio of total channel length to total basin area^1,6,30,63^) of the South Dakota basin (see Table 1). Figure 9b shows the relation between the percentage of PC and number of nodes removed. As seen from Fig. 9b, the fraction of the remaining connected pairs of nodes when critical nodes are removed from real river network also exhibits a power-law behavior for all six analyzed basins. The average power-law exponent ($slope=-\,1.02$; obtained from log-log plots from individual basins by averaging) for the real basins is slightly steeper than observed from synthetic basins. This observation of power-law slope of ~−1 and its origin for both synthetic and natural basins require further investigation and will be the focus of a future study.

### Critical node implications towards ecological and biological contexts

In this study, we identified critical nodes using the notion of connectivity. The reduction in the connectivity of a network is a measure of the integrity of the network. Biological and ecological communities in riverine ecosystems often occur in spatially structured habitats where connectivity directly plays a key role in their processes. Some previous works suggested that the hierarchical and branching structure of river networks can explain the observed traits of the riverine biodiversity^18^ and has been argued to potentially affect the biological diversity and productivity in riverine ecosystems^20^. For example, Carrara *et al*.^18^ suggested that constrained species dispersal on the dendritic network face higher extinction risks and heterogeneous habitats sustain higher levels of among-community biodiversity. Based on theoretical observations and long-term dataset, Terui *et al*.^19^ found that the branching complexity of riverine structure dampens the temporal variability of metapopulations and a loss of such complexity may affect resilience of the metapopulation^19^. They also suggested that scale-invariant characteristics of fractal river networks emerged as important stabilizing agent for riverine metapopulation^19^. Although the flow of water in river networks is directional, due to such biodiversity and ecological considerations, undirected group betweenness centrality was considered to detect critical nodes on the river network topology. In that sense, critical nodes identification and their locations have significant potential to understand the stability and persistence of biodiversity of meta-communities to stochastic watershed disturbances (e.g. floods, fires, droughts, and storms) and maintain ecological integrity on the landscapes as well as for quantifying relationships between patchy and heterogeneous habitat to network fragmentation across multiple spatial and temporal scales.

## Concluding Remarks

Critical nodes are a set of nodes whose deletion result in maximum fragmentation of the network. In this paper, using an optimization-based approach, we identified critical nodes on synthetic (obtained from optimal channel network model) and natural river networks to understand the vulnerability of river networks under potential external disruptions. We compared the identified critical nodes with the most central nodes. We also investigated the characteristics of sub-basins induced by the fragmentation due to removal of central and the critical nodes from the network for both synthetic and natural river networks through their network disruption metrics (such as pairwise connectivity and number of fragmentation). The main results of this study can be summarized as follows:

- We show that, in comparison to central nodes, critical nodes maximize fragmentation (measured by the number of connected node pairs) of a river network.
- The number of connected node pairs in the remaining network, i.e. pairwise connectivity, exhibits a striking power-law relationship with number of critical nodes removed. The central nodes do not show such a relationship.
- The number of sub-basins induced by the critical nodes removal show a similar linear relationship as a function of critical nodes removed for different *γ* values. On the contrary, central nodes curves show a higher variability with different *γ* values. In addition, on average, critical nodes removal results in a larger number of sub-basins compared to that in the case of central nodes.
- The river networks extracted (based on constant threshold flow accumulation value) from natural basins corresponding to different geographic regions, exhibited power-law relationship between pairwise connectivity and number of removed critical nodes with a slope similar to that observed in synthetic river networks. For instance, the average power-law slope for the OCNs was ~−0.86 whereas for the natural river basins was ~−1.02.

Our results reveal the potential for identifying critical nodes and their influence on basins and sub-basins characteristics under varying geomorphic, climatic, and anthropogenic activities.

## Methods

### Critical node detection framework

In this study, based on the graph-theoretic approach, we use linear integer programming formulation to detect exact locations of critical nodes on the river network topology, i.e., the nodes, whose removal minimizes the number of connected node pairs in the remaining graph. Specifically, we consider a slight variation of compact formulation techniques described in Veremyev *et al*.^41^. The presented framework allows to find exact optimal solutions on sparse graphs using the existing state-of-the art optimization solvers and standard computational power (desktop computer or laptop) within a reasonable time. We use Python 2.7 with Gurobi 7.5.2^64^ as an optimization solver to implement and solve the corresponding linear integer programs. Below we formally present general idea behind this formulation and the corresponding integer program in more details.

A river network is assumed to be represented by a simple undirected graph $G=(V,E)$ with a set of nodes *V* and edges *E*. The edge connecting node $i\in V$ and $j\in V$ is represented by a pair $(i,j)\in E$. Let $N(i)=\{j:(i,j)\in E)\}$ denote the neighbourhood of node *i*. Assume that up to *K* (=|*S*|) nodes in this graph are deleted as critical nodes. For any node $i\in V$, we define the indicator variable *v*_*i*_ as

3 $${v}_{i}=\{\begin{array}{c}\begin{array}{cc}1, & {\rm{i}}{\rm{f}}\,{\rm{n}}{\rm{o}}{\rm{d}}{\rm{e}}\,i\,{\rm{i}}{\rm{s}}\,{\rm{d}}{\rm{e}}{\rm{l}}{\rm{e}}{\rm{t}}{\rm{e}}{\rm{d}}\,{\rm{a}}{\rm{s}}\,{\rm{a}}\,{\rm{c}}{\rm{r}}{\rm{i}}{\rm{t}}{\rm{i}}{\rm{c}}{\rm{a}}{\rm{l}}\,{\rm{n}}{\rm{o}}{\rm{d}}{\rm{e}},\\ 0, & {\rm{o}}{\rm{t}}{\rm{h}}{\rm{e}}{\rm{r}}{\rm{w}}{\rm{i}}{\rm{s}}{\rm{e}}.\end{array}\end{array}$$

Then, for each pair of nodes *i*, $j\in V$ $(i\ne j)$, we define the indicator variable *u*_*ij*_ as

4 $${u}_{ij}=\{\begin{array}{c}\begin{array}{cc}1, & {\rm{i}}{\rm{f}}\,{\rm{n}}{\rm{o}}{\rm{d}}{\rm{e}}{\rm{s}}\,i,j\in V\,{\rm{a}}{\rm{r}}{\rm{e}}\,{\rm{c}}{\rm{o}}{\rm{n}}{\rm{n}}{\rm{e}}{\rm{c}}{\rm{t}}{\rm{e}}{\rm{d}}\,{\rm{b}}{\rm{y}}\,{\rm{a}}\,{\rm{p}}{\rm{a}}{\rm{t}}{\rm{h}}\,{\rm{i}}{\rm{n}}\,{\rm{t}}{\rm{h}}{\rm{e}}\,{\rm{r}}{\rm{e}}{\rm{m}}{\rm{a}}{\rm{i}}{\rm{n}}{\rm{i}}{\rm{n}}{\rm{g}}\,{\rm{g}}{\rm{r}}{\rm{a}}{\rm{p}}{\rm{h}},\\ 0, & {\rm{o}}{\rm{t}}{\rm{h}}{\rm{e}}{\rm{r}}{\rm{w}}{\rm{i}}{\rm{s}}{\rm{e}}.\end{array}\end{array}$$

The objective function, which quantifies the number of connected node pairs in the remaining graph, and the limit on the number of removed nodes can be expressed as ${\sum }_{i,j\in V}\,{u}_{ij}$ and ${\sum }_{i\in V}\,{v}_{i}\le K$, respectively.

Then, the problem formulation can be written in the following simple form:

5a $${\rm{minimize}}\,\sum _{i,j\in V}\,{u}_{ij}$$

5b $$\begin{array}{l}{\rm{subject}}\,{\rm{to}}\\ \,\,\,\,\,{u}_{ij}\ge 1-{v}_{i}-{v}_{j},\,\forall (i,j)\in E,\end{array}$$

5c $$\begin{array}{ll}{u}_{ij}\ge {u}_{kj}-{v}_{i}, & \forall (i,j)\notin E,k\in N(i),\end{array}$$

5d $$\sum _{i\in V}\,{v}_{i}\le K,$$

5e $$\begin{array}{ll}{v}_{i},{u}_{ij}\in \{0,1\}, & \forall i,j\in V.\end{array}$$

The key idea behind this formulation is to recursively model connectivity variables using constraints (5b) and (5c). Note that this formulation is slightly different from the one developed in Veremyev *et al*.^41^ and omits some constraints. Those constraints are not required due to the minimization nature of the problem and such formulation allows to relax variables *u*_*ij*_ to be nonnegative, as shown by Pavlikov^65^. In addition, for larger networks, we use more compact formulation which does not consider nodes with degree 1 (see Veremyev *et al*.^41^ and Pavlikov^65^ for more details).

As a final remark, we note that since all river networks analyzed here are trees and the considered critical node detection problem has been shown to be polynomial-time solvable on trees^66^, it can be solved using the polynomial-time algorithm, in contrast to the general case of graphs where this problem is NP-hard. However, we use the approach that is applicable to all types of graphs (rather than the specialized polynomial-time algorithm designed for trees) due to its simplicity of implementation and a very short computational time for all considered network instances. In addition, we point out that there are some other studies in the critical node detection area employing integer programming or other techniques which can be used to identify critical nodes in river networks^65,67–70^. However, they are either more complex or require some preprocessing procedures. For more information on critical node detection problems and solution techniques, we refer the reader to the recent survey by Lalou *et al*.^37^.
